# Tonsillectomy efficacy is comparable to the standard medical treatment in PFAPA syndrome

**DOI:** 10.1186/1546-0096-9-S1-P26

**Published:** 2011-09-14

**Authors:** G Vigo, G Martini, S Zoppi, F Vittadello, F Zulian

**Affiliations:** 1 Rheumatology Unit, Department of Pediatrics, University of Padua, Padua, Italy

## Background

PFAPA syndrome is the most common cause of periodic fever in childhood. Tonsillectomy has been recently suggested as an effective treatment but little is known about its long-term efficacy.

## Aim

To evaluate the clinical features, response to treatment and disease outcome in a large cohort of patients with PFAPA syndrome.

## Methods

We conducted a retrospective study on patients with clinical diagnosis of PFAPA syndrome followed at a tertiary center from January 1993 to August 2010. Clinical and laboratory parameters have been evaluated at onset and during the follow-up. Disease remission was considered as the absence of symptoms for at least one year. Disease outcome in patients who underwent tonsillectomy was compared to those treated with standard medical therapy (NSAIDs, prednisone).

## Results

275 patients with PFAPA syndrome were identified, 59.6% males, mean age at onset 27.9 months (range 1-132), mean duration of symptoms 40.5 months (10-122). A positive family history for PFAPA in first degree relatives was found in 86 patients (31.2%). The percentage of patients responding to tonsillectomy, 27/41 (65.8%), was not significantly different of what observed in those on standard therapy, 137/232 patients (59%) treated with just medical treatment (p=0.51). Neither the mean disease duration, in months, (39.7 vs 39.9) nor the age at remission (5.6 vs 5.5) were significantly different in both groups.

## Conclusions

In a large cohort of patients with PFAPA syndrome, tonsillectomy did not offer a significant advantage in comparison with the standard medical treatment.

